# Long term follow-up of a phase II trial of multimodal therapy given in a “sandwich” method for stage III, IV, and recurrent endometrial cancer

**DOI:** 10.1186/s40661-016-0027-4

**Published:** 2016-05-26

**Authors:** Michelle Glasgow, Rachel Isaksson Vogel, Jennifer Burgart, Peter Argenta, Kathryn Dusenbery, Melissa A. Geller

**Affiliations:** Department of Obstetrics, Gynecology and Women’s Health, Division of Gynecologic Oncology, University of Minnesota, Minneapolis, MN USA; Biostatistics and Bioinformatics Core, Masonic Cancer Center, University of Minnesota, Minneapolis, MN USA; Eastern Virginia Medical School, Norfolk, VA USA; Radiation Oncology, University of Minnesota, Minneapolis, MN USA; University of Minnesota, MMC 395, 420 Delaware St. SE, Minneapolis, MN 55445 USA

**Keywords:** Endometrial cancer, Sandwich therapy, Lymphedema

## Abstract

**Background:**

Our objective was to determine if previously reported overall survival (OS) and progression-free survival (PFS) rates are maintained long term following multimodal therapy for advanced and recurrent endometrial cancer and to assess the lymphedema rates associated with this therapy.

**Methods:**

Women with advanced-stage or recurrent endometrial cancer were recruited between 9/2004 and 6/2009 to our previously published Phase II trial. Patients received intravenous docetaxel (75 mg/m2) and carboplatin (AUC = 6) every 3 weeks for 3 cycles before and after radiation therapy. Patient outcomes were updated in July 2014. Data abstracted included presence of lymphedema, disease progression, and death. OS and PFS estimates at 5 years were calculated using Kaplan-Meier methods.

**Results:**

Of the 41 patients enrolled, 10 (24 %) had stage IIIA and 21 (51 %) had stage IIIC disease; 32 (78 %) had endometrioid histology; and 35 (85 %) completed the protocol. With a median follow-up of 5 years, 15 of 41 patients have died. The Kaplan–Meier estimate and 95 % CI for OS at 5 years was 70 % (53–82 %). Excluding the two patients with recurrent disease at enrollment, 15 of 39 patients progressed or died during follow-up. The Kaplan–Meier estimate and 95 % CI for PFS at 5 years was 66 % (48–78 %). Fifteen patients (37 %) had medical record documentation of lymphedema following treatment.

**Conclusions:**

After additional follow-up, OS and PFS estimates remain high and in-field recurrences low following “sandwich” therapy. The “sandwich” method remains efficacious for women with stage III-IV or recurrent endometrial cancer.

## Background

Endometrial cancer is the most common gynecologic malignancy in the United States with an estimated 52,630 new cases occurring in 2014 [1]. While most patients present with early stage disease and can be cured with surgery alone, survival is poor for those with advanced disease [2]. Adjuvant chemotherapy has been shown to control distant recurrence while adjuvant radiation has demonstrated control of local disease [3]. Several studies in recent years have shown that the combination of these two treatment modalities may be the most promising option for patients with advanced disease.

In 2011, we presented the results of a Phase II trial examining the use of carboplatin and docetaxel followed by radiation treatment and additional chemotherapy, according to the “sandwich” method in women with advanced stage or recurrent endometrial cancer [4]. With a median follow up of 28 months of 41 evaluable patients, the Kaplan-Meier (KM) estimates for overall survival (OS) at 1 year were 95 %, at 3 years 90 %, and at 5 years 71 %; the KM estimates for progression-free survival (PFS) at 1 year were 87 %, at 3 years 71 %, and at 5 years 64 %.

Several others have published retrospective and prospective studies supporting our conclusions that the “sandwich” method for the treatment of advanced stage endometrial cancer is effective and well tolerated (Table 1). Abaid et al. [5] reported that among 32 patients with advanced stage endometrial cancer (or early stage with high risk features, such as high grade and presence of lymphovascular space invasion) treated with the “sandwich” approach, PFS was 84 %, with a mean duration of follow-up of 18.9 months. Einstein et al. [6] examined the use of the “sandwich” method in 72 patients with both early and advanced stage uterine papillary serous carcinoma in a prospective Phase II trial; they estimated 3-year OS at 84 % for patients with early stage disease and 50 % for patients with advanced stage disease. In both of these studies, the rates of treatment completion were high (>90 %) and reported toxicity profiles acceptable. The hematologic and non-hematologic toxicities were mostly self-limited. However, the retrospective nature of these studies and their limited follow up prohibited a comprehensive assessment of long term treatment toxicities.

**Table 1:** Reported progression-free and overall survival for endometrial cancer patients treated with “sandwich” chemotherapy and radiation therapy

	Overall survival					Progression-free survival		
Lead Author, Year	N	UPSC/clear cell/mixed	Deaths	3-years OS	Median follow-up (months)	N	Recur, progression or death (N)	3-years PFS
Gehrig, 2004 [25]	9	100 %/0 %/0 %	0	100 %	38	N/A	1	N/A
^a^Lupe, 2007 [26]	33	33 %/9 %/15 %	13	55 % (2-years)	21	33	14	55 % (2-years)
Secord, 2007 [27]	51	N/A	5	91 %	36	51	13	69 %
^a^Fields, 2008 [28]	30	100 %/0 %/0 %	10	52 %	N/A	29	12	54 %
^a^Lupe, 2009 [16]	43	35 %/7 %/14 %	14	68 %	30	41	35	53 %
Secord, 2009 [29]	45	13 %/4 %/29 %	7	88 %	36	45	11	69 %
Geller, 2010 [17]	23	52 %/4 %/0 %	3	88 %	44	23	5	80 %
^a^Geller, 2011 [4]	41	9 %/NA/2 %	7	90 %	28	39	11	71 %
Abaid, 2012 [5]	32	13 %/9 %/9 %	3	N/A	19	8	8	84 %
^a^Einstein, 2012 [6]		100 %/0 %0 %		84 % (early stage); 50 % (advanced stage)	9	20	20	N/A
Dogan, 2013 [7]	11	18 %/18 %/0 %	0	N/A	18	1	5	N/A
Lan, 2013 [30]	35	N/A	4	82 %	36	35	9	62 %
^a^Geller, 2011 - updated	41	9 %/NA/2 %	15	75 %	60	39	15	71 %

*UPSC* uterine papillary serous carcinoma, *PFS* progression-free survival

^a^Prospective study

While these studies support the effectiveness of the “sandwich” method, toxicities do exist. Dogan et al. [7] examined acute toxicity following the “sandwich” approach in 25 women with stage IIIC endometrial cancer and compared their toxicities with those of women of the same stage who received chemotherapy followed by radiation alone. These authors found higher toxicity rates with more required dose reductions and treatment breaks, especially when pelvic and para-aortic lymph nodes were included in the radiation field. Published studies to date have not examined the long-term toxicities associated with the “sandwich” method. One of the most concerning toxicities is that of lymphedema, given the physical discomfort, pain, reduction in mobility, and negative impact on body image. [8–11]. Beesley et al. [9] recently reported the cumulative incidence of lymphedema after treatment for endometrial cancer in the Australian National Endometrial Cancer Study to be 13 %, consistent with previous reports which documented incidence rates between 1 and 18 %.

The primary aim of our current study is to present updated estimates of OS and PFS among women with advanced stage endometrial cancer who participated in our previously published Phase II trial [4]. Although important to review long term toxicity data such as gastrointestinal or genitourinary side effects, the symptoms associated with these side effects are often difficult to ascertain from a retrospective chart review. In this review, we focused on development of lymphedema instead of other long-term toxicities given few studies have reported lymphedema rates following multi-modality therapy.

## Methods

### Patient selection

Detailed methods for this Phase II trial are provided elsewhere [4]. Briefly, women with newly diagnosed advanced stage endometrial cancer (e.g. Stage IIIA-IVB according to the International Federation of Gynecology (FIGO) 1988 staging classification) or recurrent disease were recruited from the University of Minnesota Gynecologic Cancer Clinic and Methodist Hospital between 9/2004 and 6/2009. Patients were ineligible if they had received previous pelvic radiation or chemotherapy. Informed consent was obtained for all study participants for the original Phase II trial and University of Minnesota IRB approval (IRB#1406M51501) was granted for the follow-up medical chart review.

### Treatment regimen

Patients received 3 cycles (once weekly for 3 weeks) of IV docetaxel at 75 mg/m^2^ administered over 60 min followed by carboplatin area under the curve (AUC) of 6 administered over 30 min, prior to radiation therapy. Radiotherapy was initiated within 4 weeks of the third cycle of chemotherapy and was delivered using a 4-field technique to the pelvis. All patients received pelvic irradiation; the fields were expanded to encompass the para-aortic nodal chains for those with positive para-aortic nodes. The total dose to the pelvic isocenter was 45.5 Gy and to the center of the paraaortic nodal tissue between 43 and 45 Gy. HDR brachytherapy vaginal cuff boost was delivered for vaginal extension, cervical involvement, lower uterine segment involvement, parametrial extension or at the discretion of the treating radiation oncologist. Following radiation, three identical cycles of chemotherapy were initiated within 6 weeks of completing radiotherapy.

### Patient follow-up

Patients were evaluated before every chemotherapy cycle and weekly during radiation therapy. Adverse events were assessed and reported. After completion of treatment, participants underwent disease assessment with imaging 4–6 weeks following their final chemotherapy. Patients were evaluated for disease progression and recurrence every 3 months for 2 years and then every 6 months thereafter for an additional 3 years. Symptoms or exam findings concerning for disease progression and/or recurrence were confirmed with imaging and biopsy. Recurrences were defined as local if they occurred within the radiation field. Initial results were reported in 2011. Following approval from the University of Minnesota’s Institutional Review Board, a retrospective medical chart review was conducted May-July 2014 to obtain updated follow-up on all participants in this trial. Clinical data abstracted included: reported lymphedema (defined as any documentation of patient-reported lymphedema confirmed by physical exam following treatment), date of disease progression, site of recurrence and additional treatment, and date of death or date of last follow-up for those last known to be alive.

### Statistical methods

Patient demographic and clinical data were summarized and presented as frequencies (number, percent) or means ± standard deviations as appropriate unless otherwise noted. OS was calculated from date of study enrollment to death or was censored at date of last contact for patients still alive. PFS was calculated from study enrollment date to the date of first known progression or death or was censored at date of last contact for patients still alive; two patients with recurrent disease at the time of study entry were excluded from the analysis of this outcome. Kaplan-Meier estimates and 95 % confidence intervals (CI) at 3 and 5 years are reported [12]. Median OS and PFS are provided as well, however there were an insufficient number of deaths to accurately estimate the 95 % CIs. The rate of lymphedema was calculated as the proportion of participants in the study with documented lymphedema.

## Results

Forty-two patients enrolled in this trial between July 2004 and July 2009, however, ultimately 41 patients were evaluable as one patient withdrew prior to receiving any treatment. Table 2 describes the demographic and clinical characteristics of the study population. Of this group, ten patients had Stage IIIA disease, 21 had Stage IIIC disease, 1 had Stage IVA disease and 7 had Stage IV disease. The mean age at study entry was 59.0 ± 11.5 years, the majority had Stage IIIC endometrioid adenocarcinoma (51.2 %), and grades 2 and 3 histology were most common (41.5 % each). Other histologies included serous (9.8 %), mucinous (2.4 %), adenosquamous (7.3 %), and mixed serous and endometrioid (2.4 %). Two patients were treated at disease recurrence; the initial stages for these patients were Stage IC and IIA. Thirty-five patients completed the protocol as prescribed. Two participants were taken off protocol due to disease progression, one due to liver toxicity and three withdrew at their request.

**Table 2:** Demographic and Clinical Data for all Participants (*N* = 41)

	Number	Percent (%)
Age, years, mean (SD)	41	59.0 (11.5)
Race		
White	35	85.4
Other	5	12.2
Missing	1	2.4
Disease Status		
Primary	39	95.1
Recurrence	2	4.9
Stage		
IC (recurrence)	1	2.4
IIA (recurrence)	1	2.4
IIIA	10	24.4
IIIC	21	51.2
IVA	1	2.4
IVB	7	17
Grade		
1	7	17.1
2	17	41.5
3	17	41.5
Histology		
Endometrioid	32	78.1
Serous	4	9.8
Mucinous	1	2.4
Adeno Squamous	3	7.3
Endometrioid + Serous	1	2.4
Surgery Type		
Open	32	84.2
Minimally Invasive	6	15.8
*Missing*	*3*	
Extent of lymphadenectomy		
Any lymphadenectomy	36	92.3
Pelvic lymphadenectomy	5	13.9
Para-aortic lymphadenectomy	1	2.8
Both	30	83.3

Of the 39 patients with newly diagnosed disease, 36 patients had lymph nodes removed; five patients underwent pelvic lymphadenectomy alone, one patient underwent a para-aortic lymphadenectomy alone and 30 patients underwent both pelvic and para-aortic lymphadenectomy. The median number of pelvic lymph nodes removed was 17 (range: 0–32); the median number of para-aortic lymph nodes removed was 6 (range: 0–22). Twenty-six patients received pelvic radiation only, while eight patients received extended-field radiation. Two received incomplete radiation; one due to disease progression and one at patient request. The median dose of external beam pelvic radiation was 45.5 Gy (range = 21–60 Gy) in 25 fractions. The median length of time for completion of radiation was 38 days (15–55 days). Twenty-five patients were administered brachytherapy at a dose of 7 Gy in a single fraction to the proximal 4 cm of vagina at a depth of 0.5 cm and four patients received brachytherapy at a dose of 18 Gy in 3 fractions of 6 Gy, each prescribed to the proximal 4 cm of the vaginal surface.

**Fig. 1:**
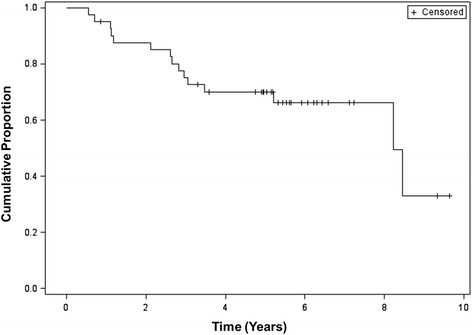
Overall survival for study participants

Updated data resulted in patients being followed for a median of 5 years (range: 0.5–9.6 years). One patient was lost to follow-up after treatment completion due to moving out of the country. There have been eight additional deaths since the initial report. Of the 41 patients enrolled, 15 patients died since the start of treatment (Fig. 1). The Kaplan–Meier estimate and 95 % CI for OS at 3 years was 75 % (59–86 %) and 5 years was 70 % (53–82 %); estimated median OS was 8.2 years.

**Fig. 2:**
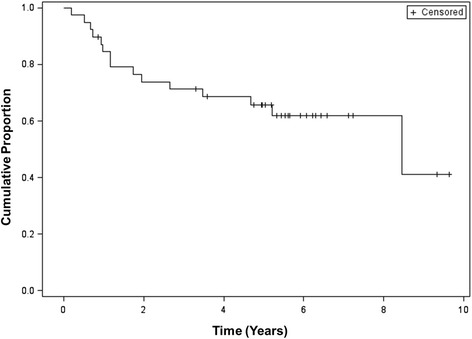
Progression-free survival for study participants. Excludes two patients who were recurrent at time of study entry

**Fig. 3:**
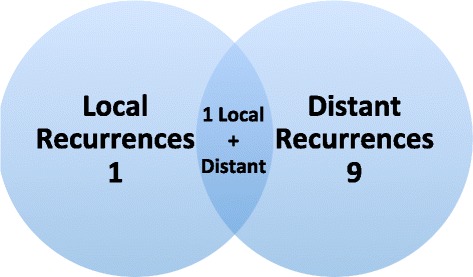
Venn diagram of recurrence sites

After excluding the two patients who enrolled at recurrence, 15 of the 39 patients progressed or died during follow-up (Fig. 2). The Kaplan–Meier estimate and 95 % CI for PFS at 3 years was 71 % (54–83 %) and 5 years was 66 % (48–78 %); estimated median PFS was 8.5 years in this subgroup. As reported in the initial manuscript, the majority of relapses occurred distantly. There was one additional recurrence to the liver since the original manuscript, occurring 56 months after study entry. In total there were two local relapses (one of which was both a local and distant failure) that occurred within the radiated field and nine distant recurrences (Fig. 3).

A total of 15 patients (36.6 %) had documented lymphedema in their medical record following treatment. Lymphedema was unilateral in nine patients and bilateral in six patients. The median time from completion of study treatment to patient report of lymphedema was 18.6 months (range 1–60 months). Stage and treatments were not consistently reported. The median time from completion of radiation treatment to patient report of lymphedema was 15 months (range 2.5–62.5 months). The extent of surgery (e.g. performance of both pelvic and para-aortic lymphadenectomy) and the radiation doses were similar between patients who developed lymphedema and those who did not. Five (33.3 %) of the patients with subsequent lymphedema were treated with extended radiation to the para-aortic chains. No relationship was observed between recurrence, location of recurrence, or surgical approach (laparotomy versus minimally invasive) and risk of subsequent lymphedema.

## Discussion

This follow-up study of women with advanced stage and recurrent endometrial cancer receiving adjuvant chemotherapy and radiation using the “sandwich” method indicates the treatment demonstrates high long-term efficacy. With a median follow-up of 5 years, estimates of OS and PFS at 5 years remain high at 70 and 66 %, respectively. Importantly, there have been no additional pelvic recurrences since the original publication, supporting the use of radiation treatment to prevent local recurrence. There has only been one additional distant recurrence during the follow-up period. This is the longest follow-up data available for this treatment modality to our knowledge.

In this prospective trial we chose to use docetaxel based on the increased neurotoxicity associated with paclitaxel in an endometrial cancer population that often has baseline neuropathy. The activity of docetaxel in endometrial cancer has been previously reported by Katsumata et al. who used docetaxel at 70 mg/m^2^ every 3 weeks in stage III, IV or recurrent endometrial cancer with an overall response rate of 31 % [13]. Günthert et al. reported response rates of 21 % in a similar previously untreated population [14]. The GOG has conducted a phase II study in previously treated recurrent endometrial cancer patients studying docetaxel 36 mg/m^2^ administered weekly every 28 days. They reported modest activity with two (7.7 %) partial responses and eight (30.8 %) with stable disease [15]. In our study, during treatment only two patients reported grade 2 neuropathy; none experienced grade 3 or 4 neuropathy. Lupe et al. reported that 31 % of their patients receiving paclitaxel and carboplatin interposed by radiation experienced grade 3 or 4 toxicity with peripheral neuropathy and neutropenia being the most commonly cited [16]. The decision to use docetaxel as opposed to paclitaxel and carboplatin over cisplatin is largely due to the grades 3 and 4 neuropathy observed in approximately 40 % of patients receiving cisplatin and paclitaxel. Endometrial cancer patients often are older and many have previously-diagnosed diabetes, therefore neuropathy tends to be a significant issue in this population. We believed that administration of docetaxel instead of paclitaxel decreased neurotoxicity allowing for completion of six total prescribed courses of chemotherapy.

In GOG 258, the primary objective was to determine if treatment with cisplatin and volume-directed radiation followed by carboplatin and paclitaxel for 4 cycles (experimental arm) reduced the rate of recurrence or death when compared to chemotherapy consisting of carboplatin and paclitaxel for 6 cycles (control arm) in patients with Stages III-IVA endometrial carcinoma. Historically for Stage III endometrial carcinoma, patients underwent surgery followed by radiation therapy. Often, systemic failure beyond treatment fields is an issue in advanced stage endometrial cancer. Alternatively, chemotherapy for this population allows for good systemic control, but poor local control. We choose to study the “sandwich” approach because in theory, sequential rather than concurrent delivery of the two treatment modalities should limit the overall toxicity and allow for maximum therapeutic dosing of both radiation and chemotherapy. Despite thorough surgical staging and cytoreduction, remaining microscopic disease can be present outside the pelvis at the time of adjuvant therapy initiation. Typically following surgery, initiation of therapy can be delayed for 2–3 weeks. If radiation therapy is given initially, as was seen in our patient population we have previously reported, it took a median of 42.5 days (range 34–62) to complete radiation therapy [17].

A multimodality regimen similar to GOG 258 was used in RTOG 9708 [18]. In RTOG 9708, pathologic requirements included grade 2 or 3 endometrial adenocarcinoma with either >50 % myometrial invasion, cervical stromal invasion, or pelvic-confined extrauterine disease. Patients received 45 Gy in 25 fractions to the pelvis along with cisplatin (50 mg/m2) on days 1 and 28. Vaginal brachytherapy was performed after the external beam radiation. Patients then went on to receive four courses of cisplatin (50 mg/m2) and paclitaxel (175 mg/m2) at 4-week intervals following completion of radiotherapy. Unlike our population that consisted only of advanced stage disease (Stages III and IV) and included serous histology, the patient population in RTOG 9708 represented an earlier stage group (stages I to IIIC); with 39 % having Stage I or II disease. The inclusion of earlier stage disease may account for the slightly higher 4-year overall survival (OS) and disease-free survival (DFS) of 85 and 81 %, respectively. In their stage III patients, the 4-year rates for OS and DFS were 77 and 72 %, respectively. In our more advanced stage population, our 4-year rates were OS and DFS was 0.70 (0.53–0.82) and 0.69 (0.51–0.81), respectively.

As we await the results from GOG 258, the protocol we follow is based on the observation that following radiation therapy there often is a treatment break before beginning chemotherapy. Theoretically, this delay could lead to disease progression prior to beginning systemic therapy in areas outside of the radiation field. Often times, if all six courses of chemotherapy are given followed by tumor directed radiotherapy, patients may have difficulty in finishing the radiation therapy due to toxicity related to the chemotherapy. Administering three cycles of chemotherapy followed by radiation allows for both modalities, with the ability to give at least some systemic therapy prior to initiating the radiation phase of the protocol.

In addition to long-term survival, we also examined the rates of lymphedema associated with the “sandwich” method. We found that 15 patients (36.6 %) were diagnosed with clinically observable lymphedema after treatment. No consistent relationship was observed between the presence of subsequent lymphedema and the extent of surgery, radiation dose, or radiation fields. Many factors may contribute to lymphedema among patients with endometrial cancer, including patient habitus, disease, and treatment-related factors. There is no consensus on the individual weight of these factors in contributing to this treatment toxicity. Several studies have demonstrated that the risk of lymphedema increases with removal of a greater number of lymph nodes. For instance, Abu-Rustum et al. [10] reported that patients who had ten or more regional lymph nodes removed were at higher risk for developing lymphedema. This association has not been found in all studies [10, 19–21]. Additionally, other studies have proposed the risk of lymphedema varies according to which lymph nodes are removed. In opposition, Yost et al. [20] found no difference in lymphedema rates in women with endometrial cancer according to the extent of lymphadenectomy (e.g. pelvic compared with pelvic and para-aortic node dissection) and Todo et al. [19] did not find para-aortic lymphadenectomy to be a risk factor for lymphedema in their review of 286 women with endometrial cancer. Hareyama et al. [22] examined the effect of preserving the circumflex iliac lymph nodes in 329 women with various gynecologic malignancies who underwent both pelvic and para-aortic lymphadenectomies. They found a lower incidence of lymphedema in the patients whose circumflex iliac lymph nodes were not removed. In our study, no significant differences were seen regarding the location of lymphadenectomy (e.g. pelvic versus pelvic and para-aortic) or in the number of lymph nodes removed in women who developed lymphedema.

The literature also suggests that radiation impacts the risk of lymphedema. Todo et al. [19] reported that whole pelvic radiation therapy was an independent risk factor for developing lower-extremity lymphedema, and this risk was reiterated by Yost et al. [20]. This association however, has not been identified in all studies. In a study of 150 women with vulvar cancer there was no increase in the risk of lymphedema in women who had previously received radiation [23]. Ryan et al. reports that in a sample population of women with lower limb lymphedema after gynecologic cancer treatment, more than half of the women with lymphedema reported having to change their daily activities as a result of lymphedema [8]. More attention is now being given to defining the effect lymphadenectomy has on the risk of lymphedema. We await the findings of GOG 244, the LEG study, a prospective longitudinal trial examining the incidence and risk factors for lymphedema and its impact on quality of life in women who have undergone radical gynecologic surgery [24].

The strengths of this study include its ability to report long-term outcomes in patients treated with the “sandwich” method. Additionally, it is the first study to describe the risk of lymphedema with this multimodal treatment. However, given the retrospective approach to identifying post-treatment lymphedema and the loss of follow-up in some patients, it is likely that the reported incidence of lymphedema is underestimated in our study. Additionally, the severity of lymphedema was not always reported, therefore limiting the utility of these data for fully counseling patients on the risk associated with this treatment modality.

## Conclusions

Our study confirms that the “sandwich” approach is a promising treatment for women with advanced and recurrent endometrial cancer, but its subsequent risk of lymphedema is not negligible. It is important to recognize this risk and to counsel patients appropriately, especially as studies have shown that lymphedema can have a significant negative impact on a patient’s quality of life after cancer treatment [20]. As the medical community develops better treatment protocols for patients with gynecologic cancers, which will hopefully lead to improved prognoses, it is also important to treat and care for conditions that may result from these cancer treatments. Studies that focus on lymphedema and other long term sequela of treatments in gynecologic cancer patients are necessary to estimate the true prevalence of these unwanted side-effects. We are currently prospectively collecting lymphedema risks and incidence in our endometrial cancer patients being treated in the “sandwich” method. This information will improve our ability to counsel patients on long-term risks of therapy and hopefully, in the future, will inform us on methods of prevention.
